# Switch of biotherapies in patients with juvenile idiopathic arthritis: analyses of the JIR cohort data

**DOI:** 10.1186/1546-0096-12-S1-P135

**Published:** 2014-09-17

**Authors:** Maryam Piram, Anne Maes, Anuela Kondi, Natalia Cabrera, Florence Aeschlimann, Carine Wouters, Gerald Berthet, Etienne Merlin, Daniela Kaiser, Laetitia Higel, Elvira Cannizzaro, Annette von Scheven-Gete, Samuel Roethlisberger, Andreas Woerner, Alexandre Belot, Michael Hofer, Isabelle Kone-Paut

**Affiliations:** 1 Pediatric Rheumatology, Kremlin-Bicêtre, France; 2 Pediatric Rheumatology, Leuven, Belgium; 3 Pediatric Rheumatology, Lyon, France; 4 Pediatric Rheumatology, Zurich, Switzerland; 5 Pediatric Rheumatology, Aarau, Switzerland; 6 Pediatric Rheumatology, Clermont-Ferrand, France; 7 Pediatric Rheumatology, Lucerne, Switzerland; 8 Pediatric Rheumatology, Strasbourg, France; 9 Pediatric Rheumatology, Romande, CHUV, University of Lausanne, Lausanne, Switzerland; 10 Pediatric Rheumatology, UKBB, Basel, Switzerland; 11 Pediatric Rheumatology Romande, HUG, University of Geneva, Geneva, Switzerland

## Introduction

Biologic treatments have been introduced for Juvenile Idiopathic Arthritis (JIA) treatment in 2000, and have substantially improved the global prognosis of all disease subtypes. However not all patients respond to one biologic and therapeutic effect of one drug may decrease with time.

## Objectives

We aimed to analyse, in current practice, the switch to another biologic, and the drug survival for treatment according to all JIA subtypes

## Methods

Patients included in the juvenile inflammatory rheumatism (JIR) cohort, including ten Swiss, French and Belgian centers for pediatric rheumatology, were analyzed retrospectively.

## Results

529 JIA patients, sex ratio: 0.5 (175M/354F), aged between 1 to 22 years at the onset of biologic treatment (mean 10.4 sd 4.4) were analysed. Their diseases were as follows: enthesitis-related arthritis (ERA: 19%), oligoarthritis (Oligo: 28%), polyarthritis (Poly: 27%), psoriatic arthritis (Pso: 5%), systemic-onset arthritis (SoJIA: 17%), and undefined (4%). Anti-TNFs were the most frequent biologics used as first line: Etanercept for 66.5% (less frequent for So: 47%), adalimumab for 13.4% and infliximab for 10%. The first biologic remained the only one in most cases (66%). The reasons to stop these treatments were: lack of efficacy for 12% (63), complete remission for 11% (57) and side effect for 7%. The second line biologic the most used was for SoJIA: anakinra (28%) and tocilizumab (24%) whereas adalimumab was the most frequent for other JIA subtypes. Tocilizumab was the top 3rd line for SoJIA (32%), and abatacept for the other subtypes. The mean drug survival in months at the date of inclusion was: 26 for etanercept, 23 for adalimumab, 12 for infliximab, 23 for anakinra, 22 for tocilizumab, 13 for abatacept but half of patients (n=265) were still on treatment at inclusion in the JIR cohort. The mean drug survival was not influenced by JIA subtype. We found a significant difference in patients having received more than two biotherapies, between SoJIA and Poly (25%) compared to other JIA subtypes (5%).

## Conclusion

Etanercept was the first biologic treatment used whatever the JIA subtype, in this retrospective study that includes patients treated since more than 10 years. Two third of cases were still treated with the first biologic after at the time of inclusion.

## Disclosure of interest

None declared.

